# Supplementary figures and images for: Metatranscriptome Analysis of the Vaginal Microbiota Reveals Potential Mechanisms for Protection against Metronidazole in Bacterial Vaginosis
Source: mSphere. 2018 Jun 6;3(3):e00262-18. doi: 10.1128/mSphereDirect.00262-18 (PMC5990888; doi:10.1128/mSphereDirect.00262-18)

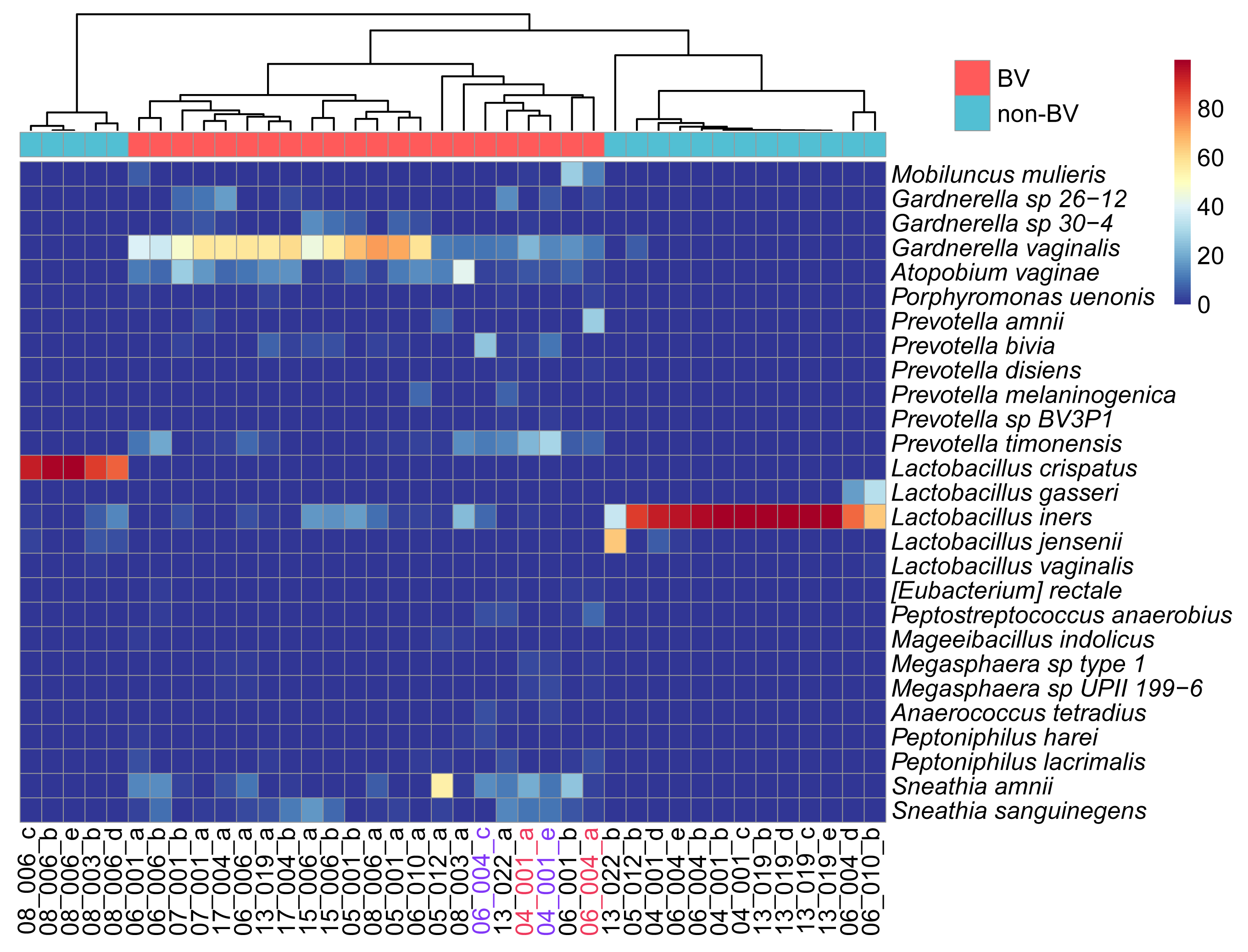

Supplement: FIG S1 [file sph003182563sf1.tif]
